# Alpha defensin immunoassay is more effective for ruling out rather than diagnosing periprosthetic joint infection (PJI): a prospective cohort study

**DOI:** 10.1186/s42836-025-00337-8

**Published:** 2025-10-08

**Authors:** Mohammad Kamal Abdelnasser, Ayat Bakhet, Amal Hosni, Dalia Tarik Kamal, Osama Bakr Osman, Mohammed Anter Abdelhameed, Mohamed MA Moustafa

**Affiliations:** 1 Orthopedic Department, Assiut University Hospital, Assiut, 71515 Egypt; 2 Clinical Pathology Department, Assiut University Hospital, Assiut, 71515 Egypt

**Keywords:** Alpha defensin, Periprosthetic joint infection (PJI), Total joint arthroplasty, Biomarker

## Abstract

**Background:**

Accurate and timely diagnosis of periprosthetic joint infection (PJI) is of utmost importance. Although synovial alpha-defensin has shown potential as a biomarker, recent studies have questioned its additional benefit over traditional synovial biomarkers and advised against its routine use. The primary objective is to evaluate the diagnostic accuracy of the alpha-defensin immunoassay in PJI. Secondary objectives include comparing its diagnostic accuracy against traditional biomarkers and assessing our results in the context of existing research to provide a solid perspective on its clinical utility.

**Methods:**

This is a prospective cohort study. Synovial samples were obtained at the time of revision arthroplasty or from painful arthroplasties. A complete laboratory workup was performed, including CBC, ESR, CRP, WBCs count. Synovial samples were analyzed for leucocytic count, PMN percentage, leucocyte esterase, and alpha-defensin immunoassay. Culture and sensitivity, and histopathology were also done. Patients who met the inclusion criteria were classified into septic and aseptic according to MSIS criteria.

**Results:**

Ninety joints met our inclusion criteria. Alpha-defensin immunoassay was positive in 36 joints and negative in 54 joints, with 1 false positive and 3 false negatives, resulting in a sensitivity of 92.11% (95% CI, 78.62–98.34%), a specificity of 98.08% (95% CI, 89.74–99.95%), positive predictive value (PPV) of 49.43% (95% CI, 12.28–87.22%), negative predictive value (NPV) of 99.84% (95% CI, 99.52–99.94%) and diagnostic accuracy of 97.96% (95% CI, 92.48–99.78%). The optimal cutoff was 9.2, and the area under the curve (AUC) was 0.945.

**Conclusions:**

While the alpha defensin immunoassay is not recommended to be used routinely as a screening method for PJI, its high specificity and NPV make it a valuable addition to traditional blood and synovial parameters in the diagnosis of complex hip and knee PJI, particularly for ruling out infection.

## Introduction

Periprosthetic joint infection (PJI) is a devastating complication of total joint replacement. It accounts for approximately 25% of failed TKA cases, 15% of failed THA cases [1, 2]. With the projected rise in the volume of primary and revision joint replacement surgeries [3–5], the incidence of periprosthetic joint infection (PJI) is also expected to increase. In addition to the economic burden that it poses on the healthcare system, PJI is associated with significant morbidity and mortality [6] [7]. In a cross-sectional study, Reinhard et al. [8] reported a 3.5% overall mortality rate among patients with PJI. Therefore, accurate and timely diagnosis of periprosthetic joint infection (PJI) is crucial for effective management and improved patient outcomes.

PJI diagnosis is multifactorial, requiring a combination of clinical signs, serum and synovial biomarkers, tissue histopathology, and culture results [9, 10]. By combining the aforementioned parameters, different groups have proposed different definitions for PJI, including the Musculoskeletal Infection Society (MSIS) 2013 [9], International Consensus Meeting (ICM) 2018 [11], Infectious Diseases Society of America (IDSA) 2013 [12], and the European Bone and Joint Infection Society (EBJIS) 2021[13].

Recently, new methods such as synovial next-generation sequencing [14, 15] and the use of artificial intelligence [16] have emerged as promising tools in the diagnosis of PJI. Among the synovial biomarkers, alpha-defensin has shown promise in diagnosing PJI, with several meta-analyses [17, 18] reporting it as having the highest odds ratio for PJI compared to other tested synovial biomarkers. Therefore, Alpha defensin was added to the ICM definition for PJI in 2018 [11].

Despite the early promising results of Alpha-defensin, with a sensitivity and specificity 100% for both the ELISA and lateral flow test [19–22], several recent studies yielded inferior results for both methods, especially lower sensitivity approaching 50% [23] and 67% [24].

Therefore, many studies have questioned the added value of alpha defensin compared to traditional and more cost-effective synovial biomarkers, such as leucocytic count and PMNs percentage, recommending against its routine use [25–28]. Similarly, several studies have reported no significant advantage of alpha-defensin over leucocyte esterase (LE), despite early reports that demonstrated superiority of alpha-defensin over Leucocyte esterase for diagnosis of PJI [19]. Li et al. [28], in a retrospective study, demonstrated that it is unnecessary to combine alpha defensin with other synovial biomarkers to diagnose PJI. Therefore, our prospective study aimed at determining whether the routine use of alpha defensin is still recommended or not.

This study's primary objective is to determine the diagnostic accuracy of the alpha defensin immunoassay in PJI. The secondary objectives are to compare its diagnostic accuracy to other biomarkers like serum CRP, ESR, synovial PMN percentage, WBC count, and LE. We hypothesize that while alpha-defensin is not recommended for routine use, it can be valuable in diagnosing complex cases of PJI.

## Patient and methods

This is a prospective cohort study conducted on patients with suspected periprosthetic joint infection PJI at the Orthopedic department of Assiut University Hospital, Egypt. After IRB approval was obtained (IRB no. 17200269), patients who met the inclusion criteria provided a written informed consent before inclusion in this study. The inclusion criteria were as follows:

1. Aspiration performed for painful arthroplasty (THA, TKA, hemiarthroplasty) due to suspected infection without radiographic signs of loosening.
2. Aspiration during revision TKA or THA (both septic and aseptic).
3. Sufficient synovial fluid available for testing.
4. Sufficient clinical and laboratory data for classification using the MSIS criteria for PJI.

Patients with inflammatory conditions, prior antibiotic administration, blood contamination of the sample, or metallosis were not excluded. However, patients with insufficient sample volume (< 3 mL), dry taps, and those with acute PJI (< 4 weeks from the index operation) were excluded.

**Fig. 1:**
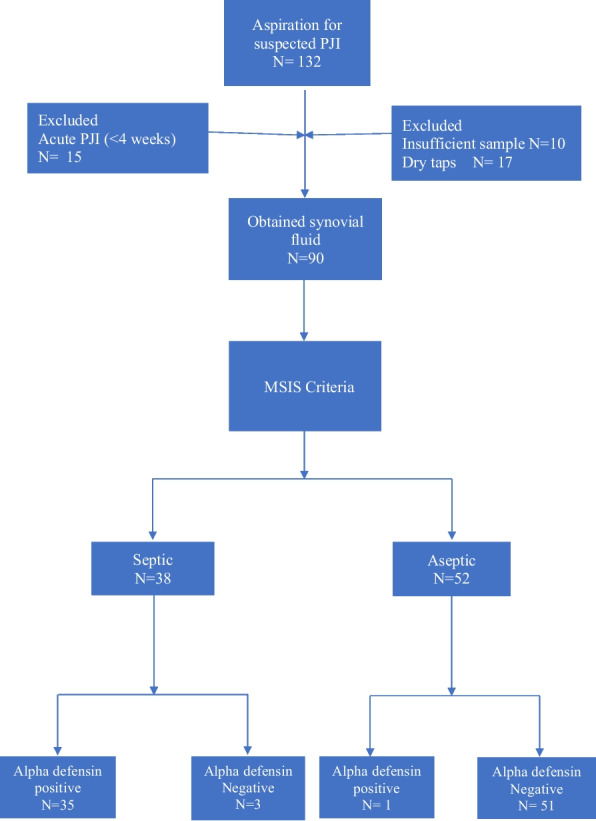
Patient flowchart

From January 2020 to September 2022, 132 joints were assessed for possible PJI. Fifteen joints were excluded for being less than 4 weeks after the index operation, and 27 joints because of insufficient synovial sample or dry taps, leaving 90 joints eligible for the study (Fig. 1). Table 1 demonstrates the basic demographics for the studied sample.

**Table 1:** Basic demographics

Parameter	N (%)
The mean age was 57.3 + 13.1 years	
**Sex**	
Male	45 (50%)
Female	45(50%)
**Site and side**	
Right hip	40 (44.4%)
Left hip	29 (32.2%)
Right knee	9 (10%)
Left knee	12 (13.3%)
**Initial diagnosis**	
Aseptic loosening	36 (40%)
Septic loosening	25 (27.7%)
Instability	8 (8.8%)
Aspiration for painful TKA, THA, or hemiarthroplasty	21(23.3%)
**MSIS definition**	
Infected	38 (42.2%)
Not infected	52 (57.8%)

As alpha defensin was interpreted in the 2018 ICM definition, the 2011 MSIS criteria were used to serve as the gold standard for PJI diagnosis [29].

A comprehensive laboratory workup, including complete blood count (CBC), erythrocyte sedimentation rate (ESR), C-reactive protein (CRP), and white blood cell count, was performed for all patients. Synovial fluid samples were obtained either by needle aspiration or during revision surgery. Samples obtained from painful TKAs were obtained by needle aspiration, whereas samples obtained from painful THAs were obtained by needle aspiration under image intensifier. All aspirations were done by an experienced arthroplasty surgeon under complete aseptic conditions. Skin preparation with antiseptic solutions (e.g., chlorhexidine-alcohol) was meticulously performed prior to sample collection. At revision surgery, samples were obtained just before arthrotomy to decrease blood contamination of the sample. Moreover, multiple tissue specimens were obtained intraoperatively and sent for histopathological examination. Synovial samples were sent for leucocytic count, polymorphonuclear leukocyte percentage (PMN%), leucocyte esterase (LE), alpha-defensin immunoassay, microscopic examination, and culture and sensitivity testing. Extended cultures were performed for 2 weeks if the initial culture was negative. We aimed to target a broad range of bacterial pathogens commonly implicated in PJI, including both highly virulent organisms (e.g., Staphylococcus aureus, Streptococcus species) and low-virulence organisms (e.g., coagulase-negative staphylococci, Cutibacterium acnes).

A standardized case report form was used to document patient history, demographic data, and clinical, laboratory, microbiological, and histopathological information. Synovial fluid samples were immediately sent to the lab after aspiration. All tests were conducted in our university hospital laboratory. Incubation conditions were maintained at 37 °C.

Samples were inoculated in culture (Blood, Chocolate, MacConkey, Sabaroud, EMB, and Mannitol). Films for Gram stain, leucocytic count, and PMN count on hemocytometer, and Leucocyte esterase tests were done without processing of the synovial fluid. The LE test was done using the Combostik10 (Spectra Group Healthcare Diagnostics, Schiffgraben, Hannover, Germany) reagent strip.

Aliquots for alpha-defensin testing, at least 1.5 mL, were centrifuged at 3000 rpm for 10 min within 2 h of collection to remove cellular and particulate content from each synovial fluid sample, and the resulting supernatant was stored − 80 °C until testing. The alpha-defensin immunoassay was performed using reagents from Glory Science Co., LTD (Area Wujiang, Jiangsu, China) and measured by standard enzyme-linked immunosorbent assay.

### Statistical analysis

Descriptive values were presented as numbers and proportions for qualitative variables. For quantitative variables, the mean and standard deviation were presented.

The diagnostic performance for the qualitative measures was assessed using cross-tabulation, while receiver operator characteristic (ROC) curves were used for the quantitative measures. An area under the ROC curve (AUC) > 0.7 was considered acceptable. The Youden Index was used to identify the optimal cut-off for the quantitative diagnostic measures. For each measure, sensitivity, specificity, positive predictive value (PPV), negative predictive value (NPV), accuracy, positive likelihood ratio (PLR), negative likelihood ratio (NLR), and diagnostic odds ratio were calculated.

Analysis was conducted using the PsychoPDA module on Jamovi software (Version 2.4) and MedCalc Software Ltd. Diagnostic test evaluation calculator (Version 22.030). All tests were bilateral, and a *P*-value of 5% is the limit of statistical significance.

## Results

According to MSIS criteria, 38 (42.2%) joints were infected, and 52 (57.8%) joints were not infected.

### Diagnostic accuracy of alpha defensin immunoassay

**Fig. 2:**
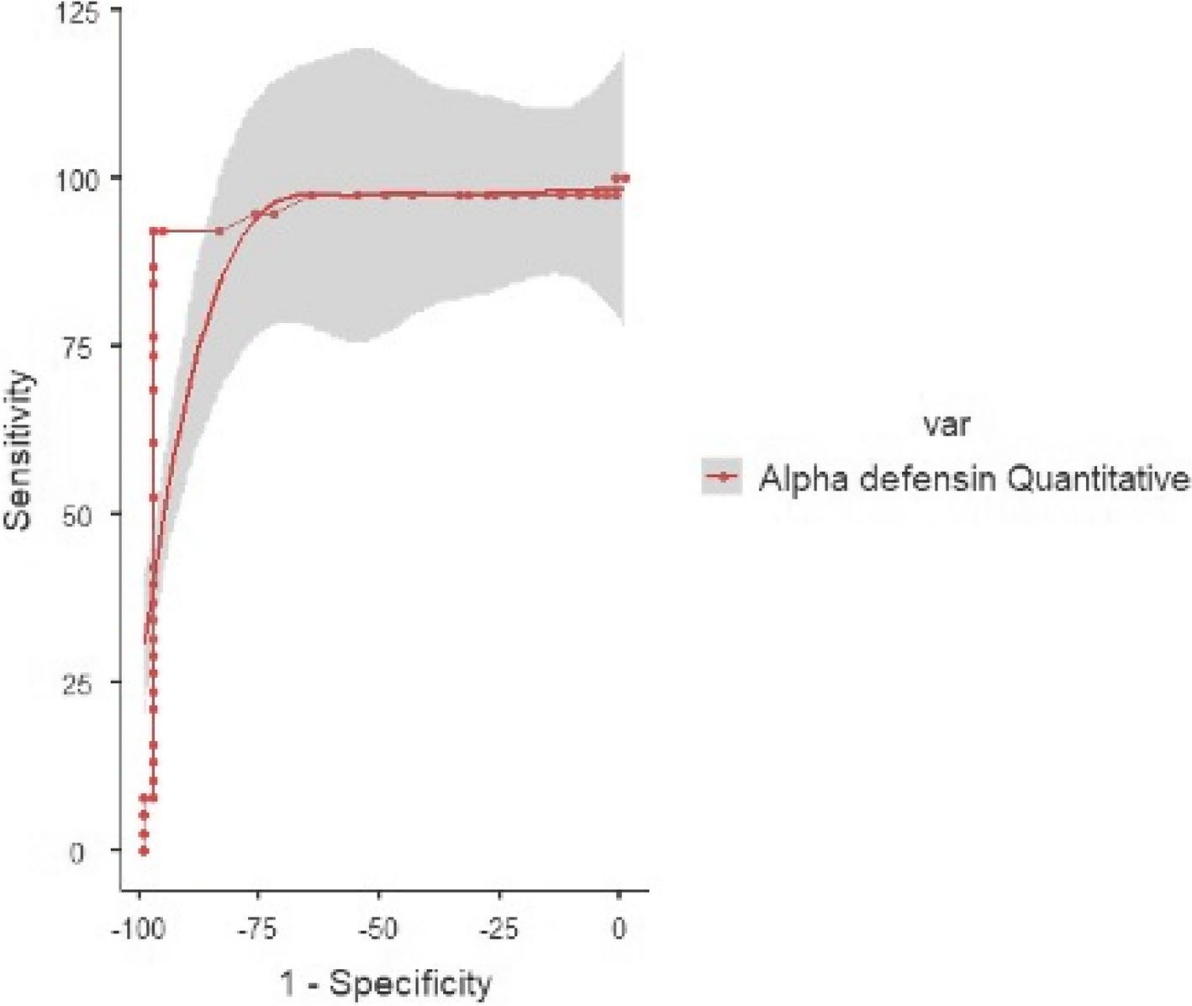
ROC curve for alpha defensin immunoassay

Alpha-defensin immunoassay was positive in 36 joints and negative in 54 joints (hip, knee) with 1 false positive and 3 false negatives resulting in a sensitivity of 92.11% (95% CI, 78.62–98.34%), a specificity of 98.08% (95% CI, 89.74–99.95%), positive predictive value (PPV) of 49.43% (95% CI, 12.28–87.22%), negative predictive value (NPV) of 99.84% (95% CI, 99.52–99.94%) and diagnostic accuracy of 97.96% (95% CI, 92.48–99.78%). The optimal cutoff was 9.2, and the area under the curve (AUC) was 0.945. Figure 2.

**Fig. 3:**
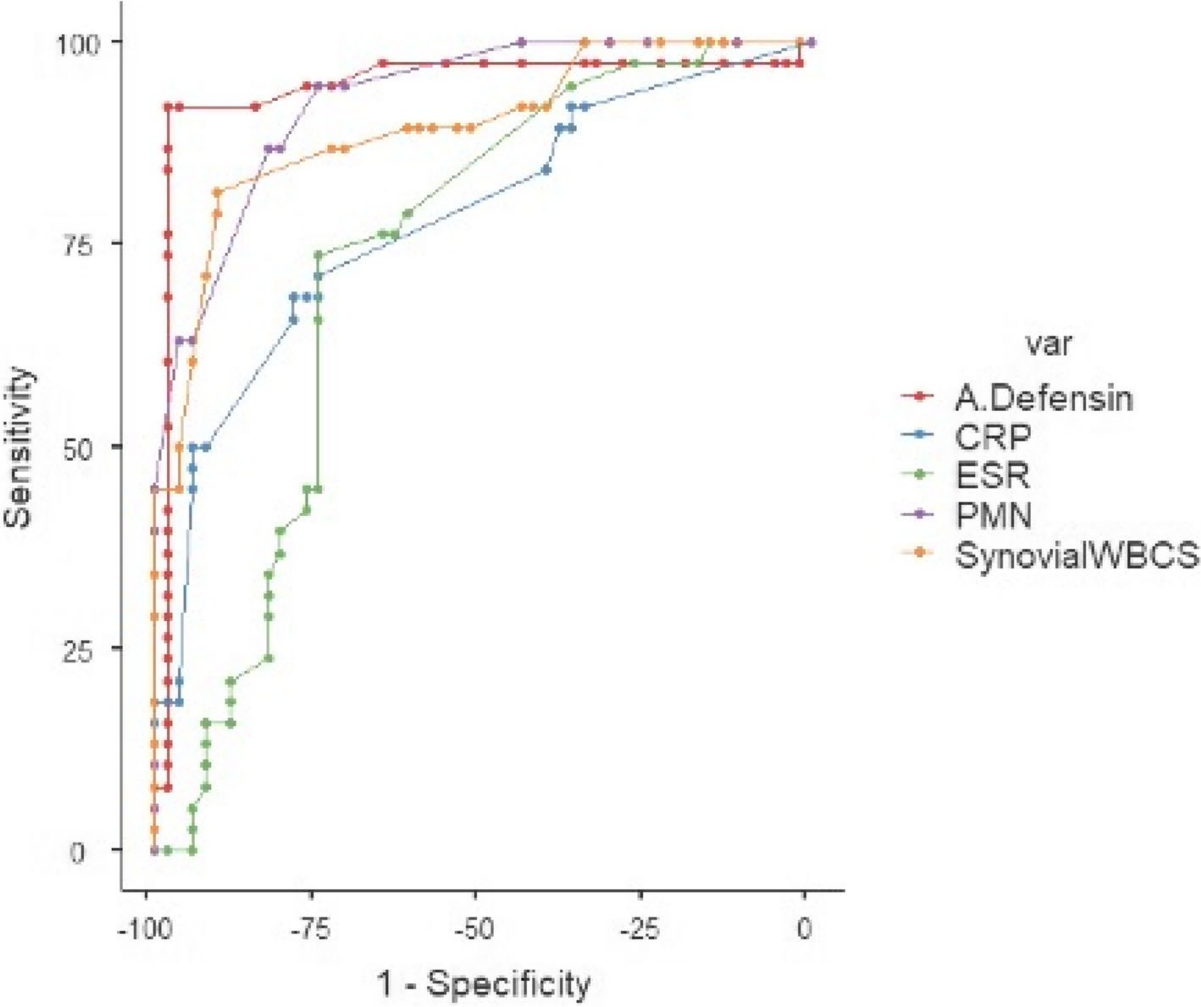
Combined ROC curves for alpha defensin immunoassay, ESR, CRP, Synovial WBCs, and PMN%

Table 2 and Fig. 3 demonstrate the diagnostic accuracy of the other serum, synovial parameters, and culture, along with the alpha defensin Immunoassay. Table 3 elaborates the comparison of the different synovial markers (including combined synovial WBC + PMN%) and culture against the alpha defensin. For all other comparisons, p-values are not statistically significant at *P* < 0.05, although the specificity of PMN% is significantly lower than that of Alpha Defensin (*P* = 0.00057).

**Table 2:** Diagnostic parameters of blood and synovial markers compared to the alpha defensin immunoassay

	**ESR**	**CRP**	**Synovial WBCs**	**PMN%**	**Leucocyte Esterase**	**Culture**	**Alpha-defensin immunoassay**
Sensitivity (95% CI)	73.68 (56.90–86.60) %	68.42 (51.35–82.50) %	81.58 (65.67–92.26) %	94.74 (82.25–99.36) %	76.09 (61.23–87.41) %	89.19 (74.58–96.97) %	92.11 (78.62–98.34) %
Specificity (95% CI)	75 (61.05–85.97) %	78.85 (65.30–88.94) %	90.38 (78.97–96.80) %	75 (61.05–85.97) %	93.18 (81.34–98.57) %	90.57 (97.34–96.87) %	98.08 (89.74–99.95) %
Accuracy (95% CI) *	74.97 (64.73–83.51) %	78.64 (68.73–86.58) %	90.21 (82.12–95.47) %	75.39 (65.19–83.87) %	92.84 (85.42–97.21) %	90.54 (82.53–95.70) %	97.96 (92.48–99.78) %
PPV (95% CI) *	5.67 (3.49–9.09) %	6.19 (3.61–10.43) %	14.76 (6.91–28.77) %	7.18 (4.58–11.08) %	18.55 (7.02–40.73) %	16.17 (7.68–30.92) %	49.43 (12.28–87.22) %
NPV (95% CI) *	99.29 (98.77–99.59) %	99.19 (98.68–99.5) %	99.59 (99.19–99.79) %	99.86 (99.45–99.96) %	99.48 (99.13–99.69) %	99.76 (99.39–99.9) %	99.84 (99.52–99.94) %
PLR	2.95	3.23	8.48	3.79	11.16	9.45	47.89
NLR	0.35	0.4	0.2	0.07	0.26	0.12	0.08
Diagnose OR	8.43	8.08	42.40	54.14	42.92	78.75	598.63

^*^NB: CRP: C-Reactive Protein, ESR: Erythrocyte sedimentation rate, PMN%: polymorphonuclear leucocyte Percentage, CI: confidence interval, PPV: positive predictive value, NPV: negative predictive value, PLR: positive likelihood ratio, NLR: negative likelihood ratio, OR: odds ratio

**Table 3:** Different Synovial markers and culture in comparison to the alpha defensin Immunoassay

	**Sensitivity (%) (95%CI)**	***P*-Value**	**Specificity (%) (95%CI)**	***P*-Value**
Alpha Defensin	92.11 (78.62–98.34) %		98.08 (89.74–99.95) %	
Synovial WBCs	81.58 (65.67–92.26) %	0.175	90.38 (78.97–96.80) %	0.093
PMN%	94.74 (82.25–99.36) %	0.644	75 (61.05–85.97) %	0.00057
Combined Synovial WBCs + PMN%	99.03 (94.60–99.83)	0.077	97.60 (92.41–99.27)	1.000
Leucocyte Esterase	76.09 (61.23–87.41) %	0.059	93.18 (81.34–98.57) %	0.169
Culture	89.19 (74.58–96.97) %	0.692	90.57 (97.34–96.87) %	0.093

## Discussion

This study demonstrated a sensitivity of 92.11% for the Alpha-defensin immunoassay, which is lower than early studies that reported nearly 100% sensitivity [19, 20] and higher than more recent studies that reported as low as 50% [23] 78.2% [30]. The specificity in our study was 98.08% which was comparable to that reported in other studies (Table 4). The positive predictive value (PPV) was 49.43%, which is markedly lower than the values reported in other studies, while the negative predictive value (NPV) was 99.8%, higher than those reported in other studies. (Table 4).

**Table 4:** Results of alpha-defensin immunoassay and lateral flow in the literature

	**Method**	**Sensitivity**	**Specificity**	**Positive Predictive Value**	**Negative Predictive Value**	**Positive Likelihood Ratio**	**Negative Likelihood Ratio**
Deirmengian et al., 2014 [17]	Immunoassay	100%	100%	NR	NR	NR	NR
Deirmengian et al., 2014 [28]	Immunoassay	97.3%	95.5%	NR	NR	NR	NR
Deirmengian et al., 2015[16]	Immunoassay	100%	100%	NR	NR	NR	NR
Frangiamore et al., 2016 [7]	immunoassay	100%	98%	NR	NR	54	0
Sigmund et al., 2018 [20]	Immunoassay	50%	98%	92%	81%	24.50	0.51
Bonanzinga et al., 2016 [32]	Immunoassay	97%	97%	88%	99%	NR	NR
Kleiss et al., 2019 [27]	Immunoassay	78.2%	96.6%	89.6%	92.2%	NR	NR
Bingham et al., 2014 [18]	Lateral flow	100%	95%	NR	NR	NR	NR
Gehrke et al., 2018 [19]	Lateral flow	92.1%	100%	100%	95.2%	NR	NR
Kasparek et al., 2016 [21]	Lateral flow	67%	93%	80%	87%	9.33	0.36
Okroj et al., 2018 [29]	Lateral flow	100%	68%	11%	100%	NR	NR
Renz et al., 2018 [30]	Lateral flow	84.4%	96.4%	86.4%	95.8%	NR	NR
Riccio et al., 2018 [53]	Lateral flow	85%	96.9%	NR	NR	27.2	0.2
Stone et al., 2018 [49]	Lateral flow	81.1%	95.9%	83.3	95.2	NR	NR
de Saint Vincent et al., 2018 [35]	Lateral flow	88.9%	90.6%	72.7%	96.7%	NR	NR
Miyamae 2019 [52]	Lateral flow	93%	100%	100%	96%	NR	NR
Sigmund et al., 2018 [20]	Lateral flow	46%	98%	91%	80%	22.27	0.56
Our study	Immunoassay	92.11%	98.08%	49.43%	99.84%	47.89	0.08

NR: Not Reported

The sensitivity of the Alpha-defensin immunoassay in other studies has ranged from 50% [23] to 100% [19, 20], and specificity of 95.5% [31] to 100% [19, 20]. In contrast, results for the lateral flow Alpha-defensin test have reported sensitivities ranging from 46% [23] to 100% [32] and specificities from 68%[32] to 100% [22] (Table 4).

The higher sensitivity of this study could be attributed to the use of MSIS criteria for diagnosis. Renz et al. [33] reported worse performance for the alpha defensin test when another definition for PJI was used. Using MSIS criteria, Renz et al. [33] reported a sensitivity of 84.4% and a specificity of 96.4% for alpha defensin compared to a sensitivity of 54.4% and a specificity of 99.3% when EBJIS criteria were used. Sigmund et al. [34] reported that the EBJIS definition is more sensitive for the diagnosis of PJIs compared to other commonly used definitions.

Our results are in concordance with many recent studies that highlighted the role of alpha defensin in ruling out rather than in diagnosing PJI. Bonanzinga et al. [35] reported a sensitivity and specificity of 97% for the Alpha-defensin immunoassay, with a PPV of 88% and a NPV of 99%. They suggested that the Alpha-defensin immunoassay could be valuable in diagnosing complex cases of periprosthetic joint infection (PJI). With a sensitivity of 50% and specificity of 98%, Sigmund et al. [23] reported that the alpha defensin immunoassay is a specific test, but is not sufficiently sensitive for diagnostic use. Similarly, Renz et al.[33] reported a low sensitivity and high (> 95%) specificity for the alpha-defensin lateral flow test, suggesting its use as a confirmatory test rather than a screening test. Drago et al. [36] also confirmed the high specificity of the Alpha-defensin immunoassay, highlighting its power in excluding PJI. On the other hand, they noted that elevated Alpha-defensin levels might be due to causes other than PJI. Using the lateral flow test, Vincent et al. [37, 38] reported a high NPV for Alpha defensin, which is important for ruling out infection in doubtful cases.

Kleiss et al. [30], in a prospective study of 202 joints, reported a sensitivity of 78.2% and a specificity of 96.6% for the Alpha-defensin immunoassay. They concluded that the routine use of alpha defensin is insufficient for accurate diagnosis of PJI. Similarly, Amanatullah et al. [25] and Kleeman et al. [26, 27] advised against its routine use, recommending it only in cases where other conventional synovial parameters yield equivocal results. Kleeman et al. [26, 27] also reported that Alpha-defensin does not add any additional benefit over traditional diagnostic tools for PJI, suggesting it may not be necessary to combine Alpha-defensin with common synovial biomarkers for PJI diagnosis. [28]

When comparing the results of this study to the results of alpha-defensin lateral flow in the literature, the alpha-defensin immunoassay is more accurate. Several studies have shown that the ELISA method outperforms the lateral flow test [24, 39] and is also more cost-effective [23]. The sample centrifugation used in the Alpha-defensin immunoassay might account for its higher accuracy compared to the lateral flow test [24]. On the other hand, Sigmund et al. [23] reported no difference between the two methods for confirmation of PJI, regardless of the definition of PJI used. However, the lateral flow test is rapid and can be used intraoperatively to confirm or rule out PJI.

In comparing Alpha-defensin with leukocyte esterase (LE), we observed a lower sensitivity (76.09%) and specificity (93.18%) for LE. Deirmengian et al. [19] similarly, a sensitivity and specificity of 100% for the Alpha-defensin immunoassay were reported, compared to a sensitivity of 68.8% and specificity of 100% for LE. In their meta-analysis, Chen et al. [40], found that while LE is less sensitive, it is more specific than Alpha-defensin. Other studies have reported similar diagnostic accuracy for both Alpha-defensin and LE [41–43]. Grünwald1et al [44] recommended the combined use of LE and Alpha-defensin to enhance PJI diagnostic accuracy, whereas Shohat et al. [45] found no advantage for Alpha-defensin over LE. Notably, the ELISA test for Alpha-defensin is considerably more expensive than the leukocyte esterase test strip, which costs around US$0.17 per test, while the Alpha-defensin immunoassay costs around US$760 per test [42]. However, the Alpha defensin immunoassay is cheaper than the lateral flow test [23].

In line with many studies [33, 46, 47], this study reported a higher sensitivity for PMN% (94.74%) among all tested biomarkers. However, alpha defensin was more specific. Hence, Alpha defensin can be used as a confirmatory test when leucocytic count is increased, as in inflammatory arthritis, dislocation, periprosthetic fractures, or in acute PJI [23, 33]. Ivy et al. [48] demonstrated that alpha defensin has a comparable accuracy to synovial white blood cells (WBCs) and polymorphonuclear leukocyte percentage (PMN%). However, they noted that Alpha-defensin’s accuracy was not statistically superior to the combined synovial WBC count and PMN%. Lee et al. [17], in their meta-analysis, compared the diagnostic accuracy of different synovial biomarkers and found that while several biomarkers (synovial fluid leukocyte count, PMN%, CRP, Alpha-defensin, LE, IL-6, and IL-8) showed high sensitivity, Alpha-defensin had the best overall performance with the highest diagnostic odds ratio.

We reported 3 false-negative PJI cases. In the first case, the culture yielded coagulase-negative staphylococci without sinus. In the second case, the infecting microorganism was Pseudomonas with a draining sinus. Although Deirmengian et al. [49] reported the consistency of Alpha-defensin results regardless of the virulence of the causative organism, several studies have shown that false-negative results can occur with low-virulent microorganisms such as coagulase-negative staphylococci, enterococci, and Cutibacterium acnes [30, 50, 51]. Adam et al. reported a case of a false-negative alpha-defensin result despite the presence of multiple positive intraoperative cultures [50], a finding reported by other studies. [30, 35, 52]. Stone et al. [52], reported seven false-negative alpha defensin with multiple positive cultures in six cases. The infecting microorganisms were mostly low-virulent organisms. Draining sinus was present in three cases. Loss of neutrophils and their peptides through the draining sinus might be the explanation for such an association. A finding supported by a low WBCs count in such cases.[30, 50]. In the third case, there was a sinus, but no growth was observed in the culture, and the synovial WBC count was only 1800. These findings suggest that the cutoff for alpha-defensin might need to be adjusted in cases of acute PJI, draining sinus, or low-virulent microorganisms [30, 50].

We reported one false positive alpha-defensin case. Although Plate et al. [53] noted an association between inflammatory arthritis and false-positive alpha defensin results, other studies [30, 54] found no such association. Miyamae et al. [55] reported high accuracy of alpha-defensin even in the presence of inflammatory arthritis. Similarly, Bonanzinga et al. [35] demonstrated that alpha-defensin was not affected by the presence of a systemic disease. Moreover, many studies reported that metallosis can be a cause of false positive results.[24, 31, 32, 35, 52, 56]. The only false-positive case in this study did not involve inflammatory arthritis or metallosis. Like Scholten et al. [57], we reported one case of metallosis that yielded a negative Alpha-defensin result.

Despite significant efforts to enhance PJI diagnosis, there remains a need for a novel biomarker with high sensitivity and specificity to enable rapid and accurate detection of PJI. A lot of novel serum and inflammatory biomarkers were introduced in the last two decades, such as calprotectin [58], D-Lactate [59], Interleukin-6, procalcitonin, TNF-alpha [60], D-Dimer [61], fibrinogen [62]. However, none of the novel biomarkers outperformed the old markers for the diagnosis of PJI [63]. Among the serum biomarkers, CRP and fibrinogen showed the best performance [64].

No single test for PJI is 100% sensitive and specific across all clinical scenarios, and a holistic assessment integrating clinical presentation, imaging, and a panel of laboratory markers remains the gold standard for accurate PJI diagnosis. Alpha-defensin, with its high sensitivity and exceptional specificity, serves as a powerful tool within this diagnostic algorithm. Its ability to effectively rule out PJI (high negative predictive value) and its strong positive predictive value make it an invaluable component, particularly when used in conjunction with established markers such as C-reactive protein (CRP), erythrocyte sedimentation rate (ESR), and synovial cell count. For instance, a negative alpha-defensin result can confidently rule out PJI, reducing the need for more invasive or costly procedures.

The strengths of this study include its prospective design and the availability of all parameters for the Musculoskeletal Infection Society (MSIS) criteria, with no missing data.

We did not exclude cases of inflammatory arthritis, dislocations, prior antibiotic administration, periprosthetic fractures, or metallosis. This is another robust evidence of the clinical utility of alpha defensin in these situations. However, there are many limitations. Firstly, the occurrence of dry taps (sicca punctures) in hip aspirations can significantly affect the diagnostic accuracy of periprosthetic joint infection (PJI) [65]. As a result, the sensitivity of the alpha defensin immunoassay may appear diminished in cases where fluid could not be obtained for testing. Another significant limitation is the use of MSIS criteria as the gold standard for diagnosis. Sigmund et al. [34] reported that the EBJIS criteria have higher sensitivity in diagnosing PJI compared to other definitions. Therefore, the high sensitivity results of alpha defensin in this study should be interpreted with caution. However, the specificity of alpha defensin in this study is in line with other studies. Renz et al. [33] reported nearly the same specificity for alpha defensin when using different PJI definitions. A third limitation of this study is the exclusion of acute PJI (< 4 weeks) and dry taps. We acknowledge that these populations represent challenging clinical scenarios, and their exclusion could introduce a degree of selection bias. Our decision to exclude acute PJI cases was based on the understanding that the inflammatory response and biomarker levels can be highly variable in the very early stages of infection, potentially confounding the interpretation of immunoassay results. Similarly, dry taps, while clinically relevant, often yield insufficient synovial fluid for reliable immunoassay testing, and their inclusion could lead to a high rate of inconclusive results, thereby impacting the overall study power and the clarity of our findings. We aimed to focus on a patient cohort where reliable synovial fluid samples could be consistently obtained and analyzed, allowing for a robust evaluation of the alpha-defensin immunoassay under optimal conditions. We recognize that this approach may limit the direct applicability of our findings to these specific challenging scenarios, and we recommend further research specifically designed to address the utility of alpha-defensin in acute PJI and cases with dry taps. We also acknowledge that a subgroup analysis by joint type (hip vs. knee) or infection chronicity would provide deeper insights into the performance of alpha-defensin. While our current study design and sample size did not allow for a statistically powered subgroup analysis for these specific categories, we recognize the importance of such an approach. Different joint types and varying infection chronicity can indeed influence the local inflammatory response and, consequently, the diagnostic accuracy of biomarkers. For instance, some literature suggests subtle differences in the diagnostic accuracy of PJI markers between hip and knee joints[66]. Similarly, the diagnostic utility of certain biomarkers, such as CRP, can vary significantly between acute and chronic PJI cases, with lower sensitivity observed in chronic, low-grade infections[66]. Future studies with larger cohorts specifically designed to include balanced representation across joint types and chronicity would be beneficial to perform such detailed subgroup analyses. We also agree that a larger sample size would enhance the generalizability of our results, particularly for rare PJI subtypes such as fungal or mixed infections. However, the incidence of these specific infections is inherently low, making it challenging to accrue large cohorts for these rare conditions in a single-center study. Our primary focus was on the diagnostic accuracy of the alpha-defensin immunoassay for PJI in general, where the majority of cases are bacterial. Future multicenter studies or meta-analyses pooling data from various studies would be beneficial to address the diagnostic performance in these less common PJI etiologies.

## Conclusion

While the alpha defensin immunoassay is not recommended to be used routinely as a screening method for PJI, its high specificity and NPV make it a valuable addition to traditional blood and synovial parameters in the diagnosis of complex hip and knee PJI, particularly for ruling out infection.

## Data Availability

No datasets were generated or analysed during the current study.
